# A modified sutureless repair for left pulmonary vein obstruction after catheter ablation

**DOI:** 10.1093/icvts/ivac097

**Published:** 2022-04-20

**Authors:** Katsuhiko Matsuyama, Hirotaka Watanuki, Masato Tochii, Kayo Sugiyama

**Affiliations:** Department of Cardiac Surgery, Aichi Medical University, Nagakute, Japan

**Keywords:** Catheter ablation, Pulmonary vein obstruction, Sutureless repair

## Abstract

A 52-year-old man presented with temporal haemoptysis and chest pain 6 months after radiofrequency catheter ablation for atrial fibrillation. Computed tomography revealed severe stenosis in the left upper pulmonary vein (PV) and complete occlusion of the left lower PV. A modified sutureless repair of the left PV obstruction was successfully performed. This modified procedure provides a feasible, safe and effective means of treating PV obstruction, even in cases with distal extension of stenosis.

## INTRODUCTION

The incidence of pulmonary vein (PV) stenosis following radiofrequency catheter ablation of atrial fibrillation is ∼1% [1]. Clinical symptoms of PV stenosis include dyspnoea, cough, haemoptysis, chest pain and recurrent lung infection with severe PV stenosis potentially being life-threatening. Percutaneous intervention for PV stenosis is associated with a high incidence of PV restenosis [2, 3]. Several surgical approaches have been recently described to treat this pathology [4–7]. Herein, we report a case of modified surgical repair for complex left PV stenosis and occlusion.

## CASE REPORT

**Figure 1:**
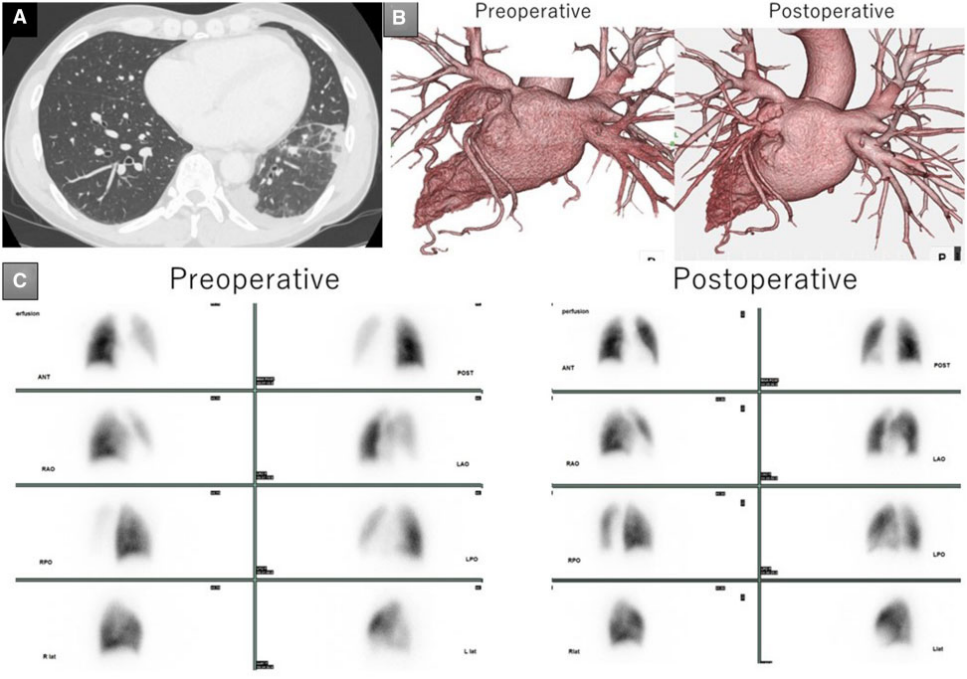
(**A**) Preoperative computed tomography showing multiple consolidated patches in the left lower lobe. (**B**) Preoperative and postoperative three-dimensional computed tomography showing severe stenosis and complete occlusion in the left upper and lower pulmonary veins, respectively. (**C**) Preoperative and postoperative scintigraphy. Preoperative scintigraphy demonstrates decreased and absent blood flow in the left upper and lower lobes, respectively. Postoperative pulmonary perfusion image improved in the left upper and lower lobes.

A 52-year-old man diagnosed with temporal haemoptysis, chest pain and low-grade fever was admitted to our hospital. He had undergone radiofrequency catheter ablation for atrial fibrillation 6 months earlier. Computed tomography revealed multiple patches of consolidation in the left lower lobe with minimal pleural effusion (Fig. 1A). Three-dimensional computed tomography revealed severe stenosis and complete occlusion in the left upper and lower PVs, respectively (Fig. 1B). Perfusion scintigraphy demonstrated decreased blood and absent blood flow in the left upper and lower lobes, respectively (Fig. 1C). Echocardiography revealed normal left ventricular function and pulmonary pressure. Failing conservative management, we proceeded with surgical repair.

**Figure 2:**
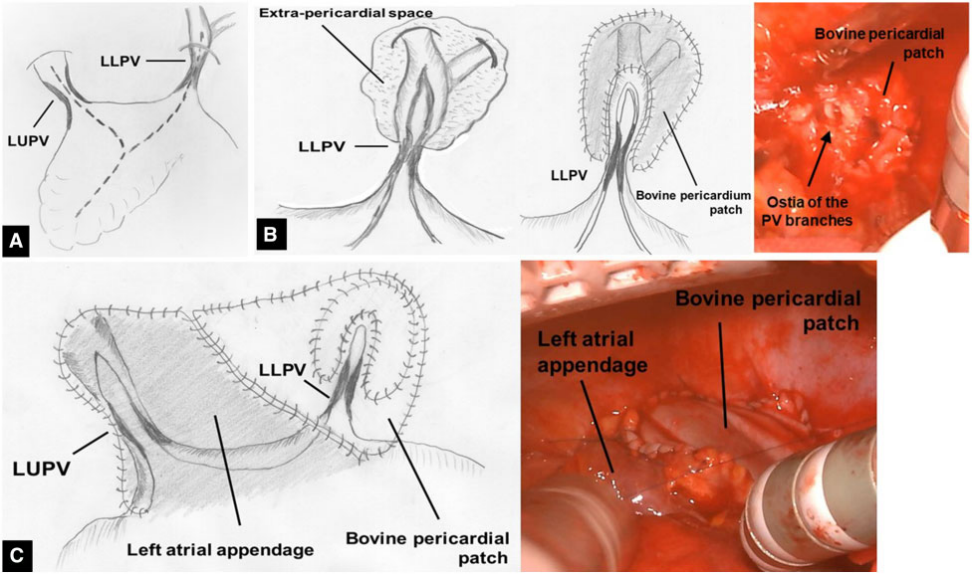
Schematic illustration and intraoperative picture of the repair technique. (A) Left appnedage incision, (B) left lower PV incision, and (C) left upper and lower PV orifices covering with a flap. LLPV: left lower pulmonary vein; LUPV: left upper pulmonary vein.

Cardiopulmonary bypass was established under general anaesthesia via median sternotomy. Cardiac arrest was observed by antegrade cardioplegic perfusion. Moderate hypothermia at a nasopharyngeal temperature of 28°C was applied to obtain a bloodless operative field, in combination with a blower using carbon dioxide. Left upper and lower PV lesions were identified after the heart was retracted to the right. The left lower PV and its branches were peripherally dissected beyond the pericardial cavity because of a lesion that was widely obstructive due to fibrous thickening. The left atrial appendage was longitudinally incised from the apex and then separately extended towards the upper and lower PVs beyond the lesion (Fig. 2A). After the left lower PV was incised longitudinally to the distal PV branch, the ostia of the PV branches were identified, and blood flow was obtained from within the lumen (Fig. 2B). No thrombi were identified in the lumen. The dissected extra-pericardial area around the left lower PV and its branches were closed with a bovine pericardial patch using a 5-0 monofilament running suture to establish venous flow from the lower PV branches directly to the pericardial cavity (Fig. 2B). Care was taken to place a needle through the PV wall at least 5 mm from the incision line. Thereafter, the left upper and lower PV orifices were covered by suturing the unpliable pericardium with a flap created using tissue from the left atrial appendage and another bovine pericardial patch (Fig. 2C). The duration of aortic cross-clamping and cardiopulmonary bypass was 103 and 155 min, respectively. The chest pain and haemoptysis subsided after surgery. Direct oral anticoagulants were commenced on postoperative Day 1. Postoperative imaging revealed successful PV reconstruction with improvement in pulmonary perfusion (Fig. 1C). The patient was discharged uneventfully on postoperative Day 10 and remains well at 4 months after surgery.

## DISCUSSION

A high rate of restenosis is associated with interventional therapy with or without stent placement for iatrogenic PV stenosis post-catheter ablation procedures (3). Surgical repair represents an alternative option stemming from congenital PV malformation surgery experience, where the sutureless technique is a well-established treatment [4–6]. This technique has the advantage of being less technique sensitive, including freedom from the requirement for direct anastomoses with the PV and avoidance of geometric distortion of PV structures.

In this case, the left lower PV required distal dissection beyond the pericardial cavity because of a widely obstructive lesion. In the sutureless technique, the extra-pericardial cavity was closed using a bovine pericardial patch. This procedure did not apply stress to the PV incision line. Moreover, the peripheral PV orifice was protected via the overlying flap. We anticipate a low risk of pseudoaneurysm formation with this technique in view of normal intracardiac pressures. Direct oral anticoagulation is planned for at least 6 months after surgery. Long-term follow-up is warranted as restenosis is a well-recognized complication. This modified procedure provides a feasible, safe and effective means of treating PV obstruction, even if the lesion extends distally to the peripheral branches.

**Conflict of interest:** none declared.

## Reviewer information

Interactive CardioVascular and Thoracic Surgery thanks Steven Hunter and the other anonymous reviewer(s) for their contribution to the peer review process of this article.
